# Gene fingerprint model for literature based detection of the associations among complex diseases: a case study of COPD

**DOI:** 10.1186/s12911-019-0738-7

**Published:** 2019-01-31

**Authors:** Guocai Chen, Yuxi Jia, Lisha Zhu, Ping Li, Lin Zhang, Cui Tao, W. Jim Zheng

**Affiliations:** 1 School of Biomedical Informatics, University of Texas Health Science Center at Houston, 7000 Fannin St Suite 600, Houston, TX 77030 USA; 2 Department of Medical Informatics, School of Public Health, Jilin University, Changchun, Jilin, 130021 China; 3 Department of Development Pediatrics, The Second Affiliated Hospital of Jilin University, Changchun, Jilin, 130041 China; 4 Department of Respiratory Medicine, The Second Affiliated Hospital of Jilin University, Changchun, Jilin, 130041 China

**Keywords:** Disease connection, Gene fingerprint model, Chronic obstructive pulmonary disease, COPD

## Abstract

**Background:**

Disease comorbidity is very common and has significant impact on disease treatment. Revealing the associations among diseases may help to understand the mechanisms of diseases, improve the prevention and treatment of diseases, and support the discovery of new drugs or new uses of existing drugs.

**Methods:**

In this paper, we introduced a mathematical model to represent gene related diseases with a series of associated genes based on the overrepresentation of genes and diseases in PubMed literature. We also illustrated an efficient way to reveal the implicit connections between COPD and other diseases based on this model.

**Results:**

We applied this approach to analyze the relationships between Chronic Obstructive Pulmonary Disease (COPD) and other diseases under the Lung diseases branch in the Medical subject heading index system and detected 4 novel diseases relevant to COPD. As judged by domain experts, the F score of our approach is up to 77.6%.

**Conclusions:**

The results demonstrate the effectiveness of the gene fingerprint model for diseases on the basis of medical literature.

## Background

The coexistence of diseases, termed comorbidity, describes the presence of multiple diseases or conditions in the same person [1]. Comorbidity is very common in clinical practice, for example, 31% of adult patients with arthritis had obesity, 47% had diabetes and 49% had heart disease in the United States in 2013–2015 (cdc.gov). Comorbidity is not a simple addition of diseases on another that independently follow their usual trajectories [2]. Due to rapid advances in genomic technologies, genetic analyses have become vital in clinical practice and research to understand the gene-disease relationships. Revealing the associations between diseases and genes as well as between diseases may help to understand the mechanisms of diseases, improve the prevention and treatment of diseases, and support the discovery of new drugs or new uses for existing drugs [3–5].

Literature in the biomedical domain, as a significant addition to experimental data, has been broadly used by researchers for the inference of gene regulatory network [6], analysis of the relationship between drugs, genes and diseases, and other biomedical research purposes. For example, researchers inferred disease-disease associations [7] from PubMed abstracts and biological pathways and used large-scale knowledge-bases such as the Online Mendelian Inheritance in Man (OMIM) to find the disease-causing genes [8, 9].

Chronic obstructive pulmonary disease (COPD) is a common respiratory disease ranked as the third leading cause of death and the second leading cause of disability in the world [10]. COPD also continues to be a major cause of morbidity and mortality in the United States. Approximately 6.5% of the U.S. adults (an estimated 15 million) have been diagnosed with COPD [11]. COPD develops through the interaction of environmental and genetic factors, and the exact etiology is still not clear. Therefore, the study of COPD is an important topic in biomedical research. Genome wide association study (GWAS) and other biomedical research found many candidate susceptibility genes for COPD, including but not limited to SERPINA1, EPHX1, GST, MMP12, TGFB1, SERPINE2, CHRNA3/5 and HHIP [12–15]. Finding additional genes and understanding their role in COPD may lead to the development of specific treatments and promote early prevention, detection and treatment.

Many experimental and quantitative researches have focused on predicting and knowledge mining of COPD genes. Using known COPD gene information, these studies identified genetic factors associated with COPD [16], discovered clinical features and genetic risk factors that overlap between COPD and asthma [17], found genetic determinants of quantitative imaging phenotypes [13], and detected a deletion affecting total lung capacity among subjects [18]. However, experimental data collection is a long and laborious process. Bioinformatics efforts, one the other hand, could speed up our understanding of the molecular mechanism of COPD. Some of these approaches have focused on mining relevant knowledge from medical literature [19–21], and building the biological pathways through visualization [22]. Novel methods such as Ontology Fingerprints [23] have been successfully used to infer active signaling pathways in cancer cells [24], to develop biological networks [25], and to help with personalized cancer therapy [26].

In this paper we report a novel approach to discover the relationships between COPD and other diseases. We also introduce a mathematical model to represent gene related diseases with a series of associated genes based on PubMed literature and Medical Subject Headings (MeSH) [27]. MeSH is an index catalogue with hierarchical structure in life sciences and used to annotate journal articles and books for different databases such as MEDLINE articles and Clinical Trials registry. Moreover, we illustrate an efficient way to reveal the implicit connections between COPD and other diseases based on this model. Our results not only confirmed known disease-disease relationships for COPD, but also identified novel diseases related to COPD. The findings build a solid foundation to understand how COPD is related to other diseases, and drugs treating these diseases could be a useful resource in treating COPD.

## Materials and methods

### Data and materials

In this study, we focused on evaluating the relationships between COPD and the other lung diseases under the Lung disease branch in MeSH (MeSH tree id C08.384). We used this approach since the relationships between COPD and most of these diseases have been well studied, providing useful evidence to evaluate our methods. Among these diseases, we ignored those not linked to any gene and used the resulting 82 diseases for the study. The publication to gene relationship was obtained from the Oct 5, 2017 version of the gene2pubmed file downloaded from the National Center for Biotechnology Information (NCBI). The PubMed citations used were last updated on Sept 28, 2017.

### Methods

**Fig. 1:**
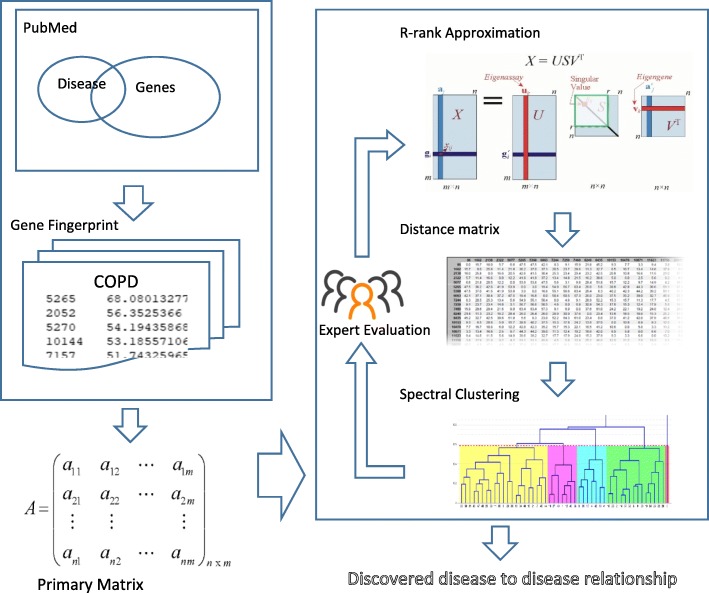
Work flow Diagram

As illustrated in Fig. 1, we first created a gene profile, named Gene Fingerprint, for each disease based on its MeSH annotations in PubMed. For the targeted diseases (i.e., all diseases under the Lung Diseases branch in MeSH), we identified an appropriate low rank approximation [28] for the primary matrix to detect novel disease-to-disease relationships through the spectral clustering algorithm [29].

#### Gene fingerprint for disease

Inspired by the development of gene Ontology Fingerprint and the success of its applications in several fields [23–25], we developed the Gene Fingerprints for a disease — a set of genes that are over-represented in the literature relevant to the disease together with the enrichment *p*-value, and thus established a mathematical model to represent the disease with a series of associated genes. However, to eliminate the possibility of propagating noise through this process, only directly co-occurring genes were taken into account, and further processing (see below) was applied to ensure implicit relationships could be detected.

The human genes to PubMed relationship was extracted from the gene2pubmed file. Disease to PubMed relationship was obtained from the MeSH indexed information in PubMed XML files, including both the downloaded baseline and update files. For every disease, hypergeometric distribution [30, 31] was applied to evaluate the significance of its association with each human gene using the relationship illustrated below:

**Table:** 

	Disease *d*_*i*_	Not Disease *d*_*i*_	
Gene *g*_*j*_	PubMed for *d*_*i*_ & *g*_*j*_*(k)*	PubMed for *g*_*j*_, not *d*_*i*_	All PubMed for *g*_*j*_*(K)*
Not gene *g*_*j*_	PubMed for *d*_*i*_, not *g*_*j*_	PubMed not for *d*_*i*_ or *g*_*j*_	All PubMed for not *g*_*j*_
	All PubMed for *d*_*i*_*(n)*	All PubMed for not *d*_*i*_	All PubMed (diseases, N)

The calculation resulted in a Gene Fingerprints for each disease. We used the absolute logarithmic scaled values of the enrichment p-value for the computation convenience in Gene Fingerprints. Therefore, a disease *d*_*i*_ can be represented as Eq. 1, where *g*_*j*_ is an associated gene, *p*_*ij*_ is the enrichment *p*-value from the hypergeometric enrichment test, *k* is the number of PubMed abstracts containing both gene *g*_*j*_ and disease *d*_*i*_, *K* is the number of PubMed abstracts containing gene *g*_*j*_, *n* is the number of PubMed abstracts for disease *d*_*i*_, *N* is the total number of PubMed abstracts for diseases and *m* is the collection of all associated genes with disease *d*_*i*_.

1 $$ {d}_i=\left\{\left({g}_j,-\log \left({p}_{ij}\right)\right)|j\in \left[1..m\right]\right\} $$

where

$$ {P}_{ij}=\frac{\left(\begin{array}{c}K\\ {}k\end{array}\right)\left(\begin{array}{c}N-K\\ {}n-k\end{array}\right)}{\left(\begin{array}{c}N\\ {}n\end{array}\right)} $$

#### Analysis of disease relationships with the Low-rank matrix approximation

Using matrix approximation for information retrieval was initially introduced for Latent semantic analysis (LSA) [32, 33] by replacing the original term-document matrix with a low-rank approximation of the origin. A typical technology to produce low-rank matrix approximations is singular value decomposition (SVD) [34]. An approximation of a matrix could be produced by replacing part of the smallest singular values on the diagonal of the scaling matrix with zeros using SVD. The logic behind the process is that with the linear transformation, the vectors for documents are rescaled towards their latent principle components in proportion to the rank of the approximate matrix [34, 35]. Through this approach, the implicit relationships among documents that do not share common terms could be discovered [33].

We created a primary matrix with rows representing diseases and columns representing genes for 82 lung diseases based on the disease Gene Fingerprint model. Genes associated with only one disease were removed in the matrix. Using this primary matrix, we then establish a disease to disease matrix measured by Spearman correlation distance based on a low-rank approximation of the primary matrix. The diseases in this matrix were clustered with the Spectral clustering algorithm to reveal relationships between diseases.

#### Model evaluations with COPD case

The diseases under the Lung diseases branch in the MeSH tree were categorized by five independent lung disease experts into three mutually exclusive groups: related to, not related to, and undefined relationship to COPD. The label of a disease was derived as follow: a disease will be marked as related if agreed by 4 or 5 experts, non-related if agreed by 3 experts or more, undefined otherwise. Among the 82 Lung diseases, 49 were marked as related, 24 as non-related, and 9 as undefined.

We used the 49 positive and 24 negative cases as training data to estimate the rank of the approximation of the matrix. The best performing matrix compared with the experts’ annotation was selected as the most efficient approximation of the primary matrix. This approximate matrix was then used to assess the relationship between the 9 undefined diseases and COPD, from which the novel associations were detected.

#### Evaluations of detected novel diseases

To evaluate the novel disease association for COPD, we analyzed the gene to gene and gene to disease relationships, as well as the associations from literature using the following methods and systems.

#### Analysis through the disease associated gene fingerprints

We assessed the contribution of the genes in the diseases’ Gene Fingerprints to the relationship between COPD and other detected diseases. We gradually removed the genes whose enrichment *p*-value were less significant than a threshold. The connections between COPD and these diseases were then re-evaluated using the filtered Gene Fingerprints.

#### Ingenuity pathway analysis (IPA™)

IPA™ [36, 37] has been widely used by the research community to explore the relationships among genes, diseases and pathways. Many results obtained from IPA analysis have been experimentally validated, indicating IPA as a credible source for analyzing these relationships. We explored the relationships between COPD and the detected diseases in IPA™ as a way to validate our findings and to provide additional insight into the mechanisms of discovered disease connections.

#### Semantic MEDLINE database (SemMedDB)

SemMedDB [38, 39] is an NIH maintained repository of semantic predications extracted using SemRep and covers all the relationship information of the medical concepts in 32 categories in MEDLINE. SemMedDB literally explains the pathway between COPD and the detected novel diseases through sematic relationships.

#### The database for annotation, visualization and integrated discovery (DAVID)

DAVID [40, 41] is an online bioinformatics resource developed by the Laboratory of Immunopathogenesis and Bioinformatics (ncifrederick.cancer.gov), which is a NCI lab located at Frederick, Maryland. It provides integrated functional annotation tools for significant gene sets obtained from genome studies. DAVID tests the enrichment of the functional annotations such as biological process, molecular function and pathway for a gene set. We used David to evaluate the Gene Fingerprint models of the diseases and the novel relationships between COPD and the detected diseases.

## Results

Using the 49 positive and 24 negative cases as training data, we identified an approximation of the primary matrix that retained 95% energy as the most efficient matrix to assess the relationships between Lung diseases. This approximation selects *r* largest eigenvalues such that their summation occupies 95% of the total eigenvalues’ summation [42] for all cases.

Due to limited amount of information provided by the small number of genes, diseases with a small Gene Fingerprints may skew the results of disease-disease connections. We evaluated the matrix approximation models by eliminating diseases with 1, 2, and 3 genes in their Gene Fingerprints. As shown in Table 1, the precision of detecting disease-disease associations is not greatly affected by eliminating diseases with 1, 2 or 3 genes in their Gene Fingerprints. However, recall is significantly changed.

**Table 1:** The performance of the model on diseases with minimum number of required genes in their Gene Fingerprints

Minimum # associated genes	Related diseases	Unrelated diseases	Undefined diseases	Detected diseases	Precision	Recall	F score
1	49	24	9	Lung Injury, Sarcoidosis-Pulmonary, Acute Lung Injury, Bird Fancier’s Lung	70.7%	59.2%	64.4%
2	47	24	9	Lung Injury, Sarcoidosis-Pulmonary, Acute Lung Injury, Bird Fancier’s Lung	71.7%	70.2%	71.0%
3	44	23	8	Lung Injury, Sarcoidosis-Pulmonary, Acute Lung Injury, Bird Fancier’s Lung, Eosinophilic Granuloma, Pulmonary Veno-Occlusive Disease, Meconium Aspiration Syndrome	70.4%	86.4%	77.6%

**Fig. 2:**
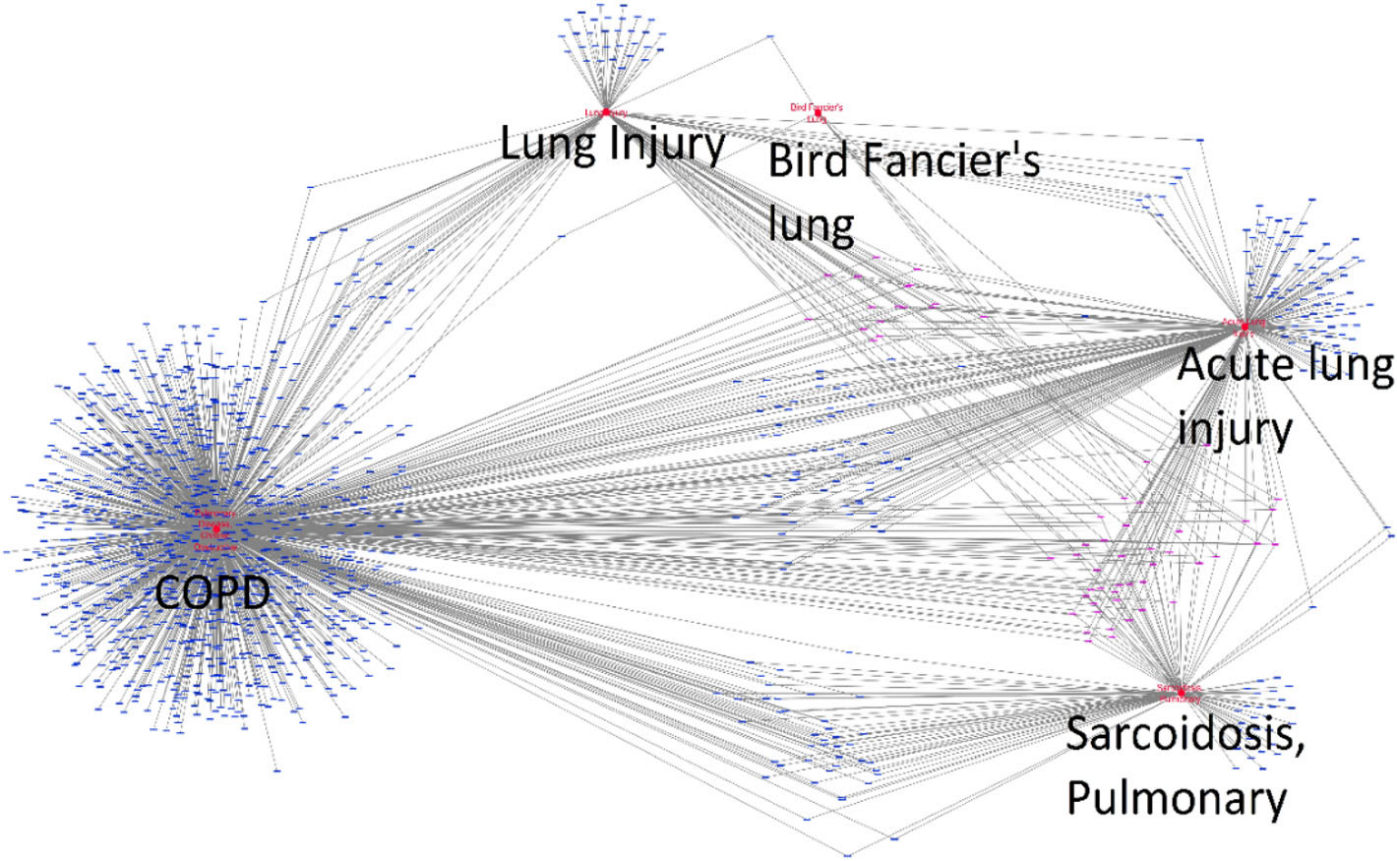
Illustration of the connections between the 5 diseases through shared genes. The genes in pink are associated with more than 2 diseases

Out of the 9 diseases with no known connections to COPD, we used our model to identify 4 that are related to COPD: Lung Injury, Sarcoidosis Pulmonary, Acute Lung Injury, and Bird Fancier’s Lung. Based on our analysis, we found that these diseases have significant connections to COPD. The newly detected connections through the genes in the Gene Fingerprints of these diseases are visualized in Fig. 2. These results indicate that our Gene Fingerprint based method can identify novel relationships between COPD and other diseases.

**Fig. 3:**
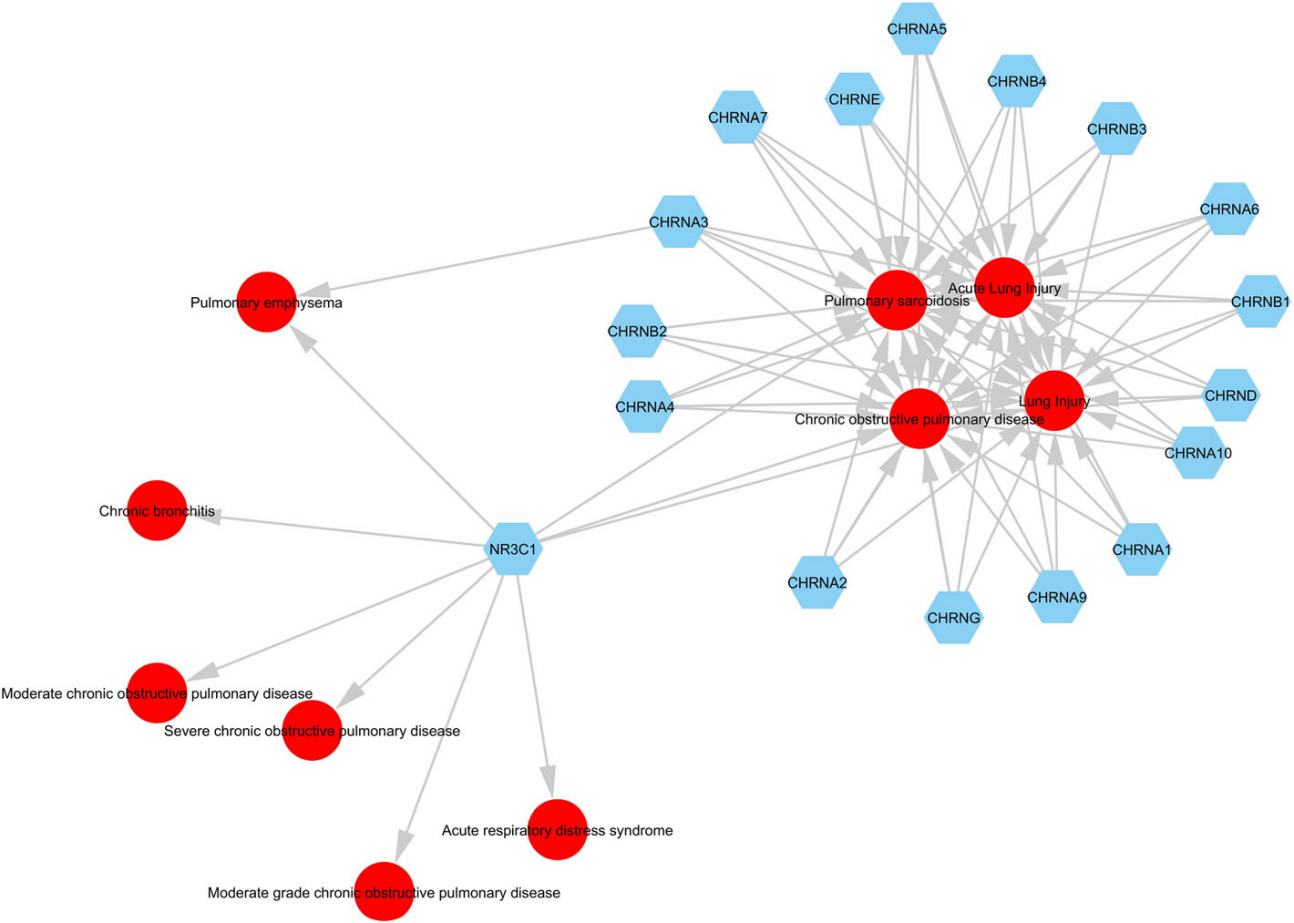
IPA identified 17 genes that affect COPD, Pulmonary Sarcoidosis, Lung Injury and Acute Lung Injury. Blue nodes represent genes and red nodes represent diseases. The four diseases discussed in Results section are located in the middle of the circle

To further understand the newly identified disease-disease relationships, we analyzed these diseases in Ingenuity Pathway Analysis (IPA™). As illustrated in Fig. 3, IPA™ identified 17 genes that affect COPD and all the novel diseases except Bird Fancier’s Lung. On the left side of Fig. 3, the gene NR3C1 is one type of glucocorticoid receptor gene located on chromosome 5q31–32 in humans. It undergoes alternative processing to produce multiple functionally distinct variants of glucocorticoid receptor (GR) [43]. GR is a target of inhaled corticosteroids that are commonly used to treat COPD [44]. Interestingly, glucocorticoids, which are a type of corticosteroids, can also be used to treat pulmonary sarcoidosis [45] and acute lung injury [46]. When corticosteroids bind to GR, the activated GR complex can up-regulate the expression of anti-inflammatory proteins in the nucleus or repress the pro-inflammatory proteins in the cytosol [47].

The 16 genes on the right of Fig. 3 all belong to the nicotinic cholinergic receptor (CHRN) family, a well-known susceptibility gene family for COPD [48]. CHRNA1–7, CHRNA9–10 are CHRN α genes, CHRNB1–4 are CHRN β genes, CHRND, CHRNE and CHRNG are CHRN δ, ε, γ genes. CHRNA3, CHRNB4 and CHRNA5 are the most recognized susceptibility genes for COPD [49]. CHRNA7 is located on the surface of immune cells. After activation, it mediates cholinergic regulation of inflammation and results in a decrease in pro-inflammatory cytokine production [50]. CHRNA7 is associated with pulmonary sarcoidosis, whose expression is significantly elevated in peripheral blood mononuclear cell in patients with pulmonary sarcoidosis compared with healthy controls [51]. Therapeutic activation of the CHRNA7-dependent nicotinic anti-inflammatory pathway represents a theoretical intervention to prevent progression of sarcoidosis [52]. CHRNA7 also plays a role in acute lung injury and is a potential target for the treatment of this disease [53]. These findings indicate that the newly detected connections between diseases and COPD are supported by common molecular mechanisms related to GR, the CHRN family and inflammation. Notably, only two out of 17 genes appear in these diseases’ Gene Fingerprints, indicating that IPA™ and the Gene Fingerprint approach are complementary and supportive of each other.

**Fig. 4:**
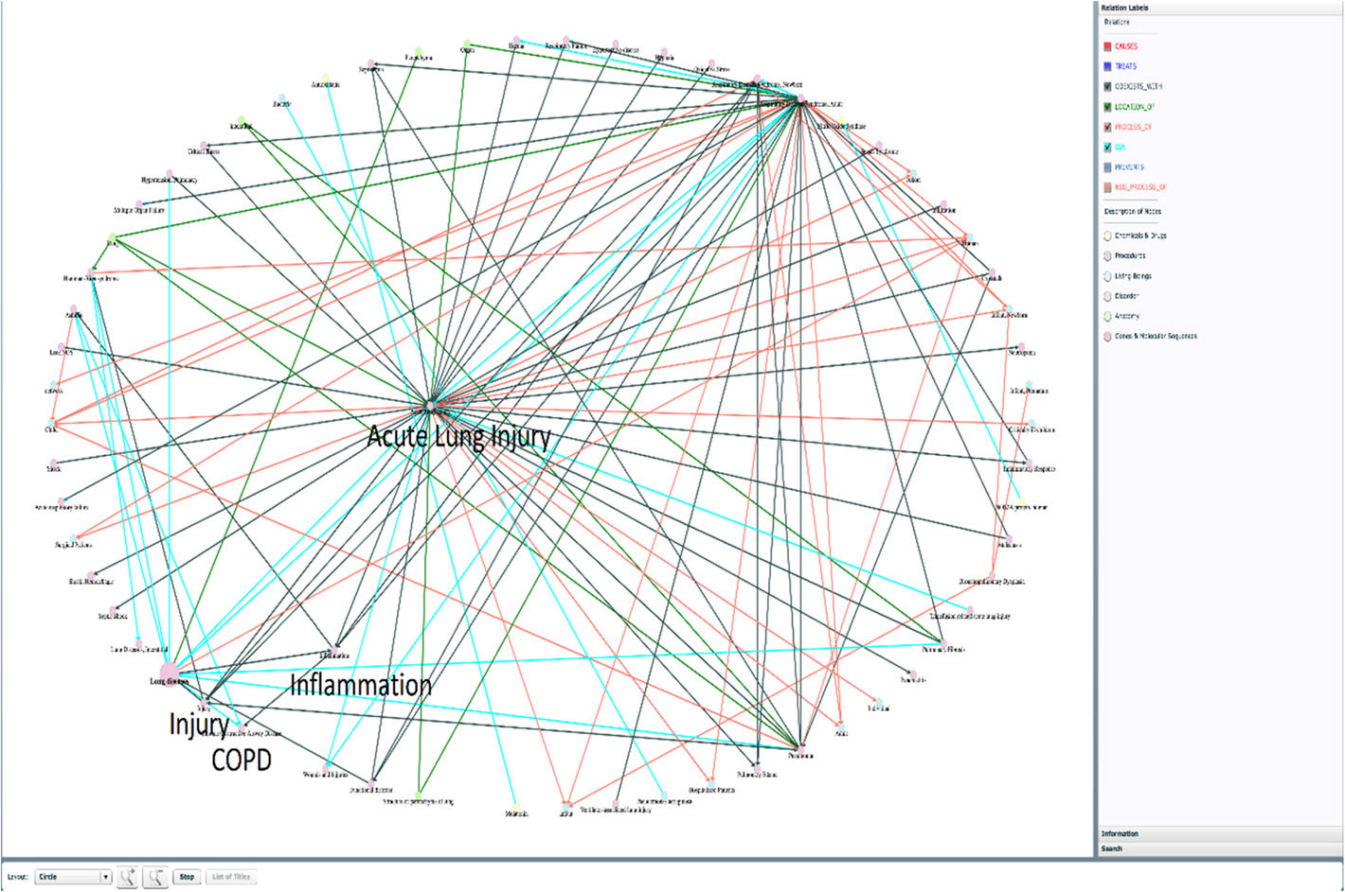
Connections between Acute Lung Injury, Lung Injury and COPD in the semantic network generated in Semantic MEDLINE

We also used the Semantic MEDLINE [38, 39] to explore the relationship among these diseases. Semantic MEDLINE is a web application that summarizes MEDLINE citations obtained from a PubMed search. As illustrated in Fig. 4, the semantic network generated from Semantic MEDLINE indicates that Acute Lung Injury coexists with Inflammation, and Inflammation coexists with COPD, which implies that Acute Lung Injury likely leads to COPD if inflammation happens. Likewise, Lung Injury similarly leads to COPD through Respiratory Distress Syndrome and Inflammation. However, Pulmonary Sarcoidosis does not have a meaningful connection to COPD in Semantic MEDLINE.

We also explored the disease relevance from highly relevant genes in the Gene Fingerprint of the diseases. One hundred and seventy two highly relevant genes were obtained from the Gene Fingerprints of the 4 novel diseases and COPD after applying an association *p*-value cutoff of 0.01, a value corresponding to the significance at the 0.01 level and 99% confidence interval.

For each disease, its highly relevant genes were analyzed by DAVID [41] to obtain the enriched KEGG pathways with a Bonferroni cutoff of 0.05. We obtained 72 significantly enriched pathways for COPD, which also includes all the 14 enriched pathways associated with Acute Lung Injury and the 4 with Lung Injury. There are 17 enriched pathways associated with Pulmonary Sarcoidosis, 12 of which are the members of the 72 pathways associated with COPD. Three of these 12 pathways also overlap with pathways associated with Acute Lung Injury, with the remaining 5 being unique to pulmonary sarcoidosis.

**Fig. 5:**
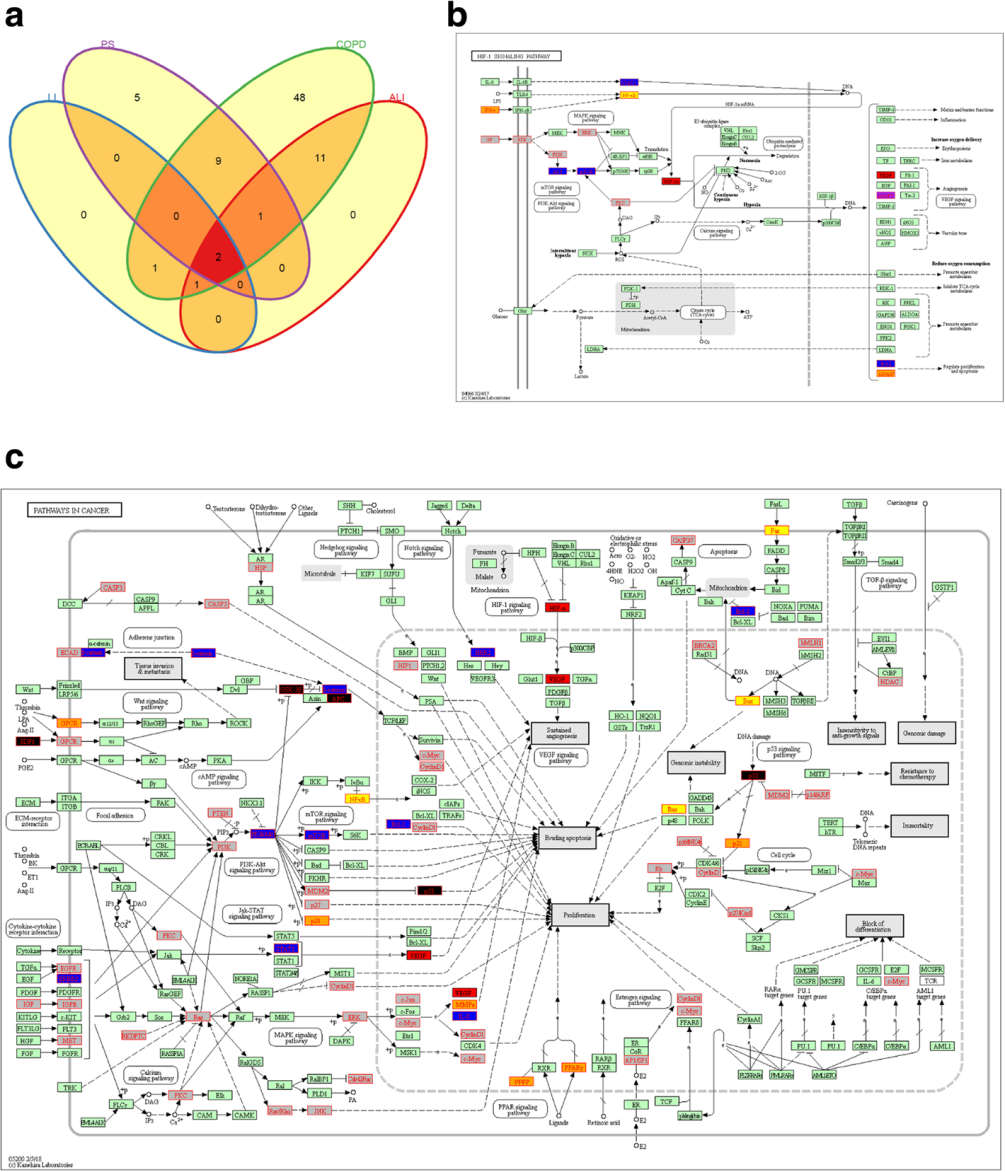
Enrichment analysis with DAVID 6.8 of the associated genes for the 4 novel diseases and COPD. Two enriched KEGG pathways shared by the four diseases. **a** Venn graph to show the number of overlapping enriched KEGG pathways among the 4 diseases. LI: Lung injury; PS: Pulmonary Sarcoidosis; COPD: Chronic Obstructive Pulmonary Disease; ALI: Acute Lung Injury. Two KEGG pathways are shared by the four diseases. **b** HIF-1 Signaling pathway. **c** Pathways in cancer. Different colors of the box represent the genes in the pathway that are shared by different diseases. Blue: ALI & COPD; red: ALI & LI & COPD & PS; yellow: ALI & COPD & PS; black: LI & COPD; purple: ALI & LI & COPD; orange: COPD & PS; gray: COPD unique except for LPAR1 which is LI unique. RELA belongs to LI & COPD, ERBB2 belongs to ALI & COPD, MMP2 and MMP9 belong to ALI & LI & COPD, LPAR1 is LI unique. Since they correspond to the same KEGG symbol with other genes, the colors are represented by the first gene in each group

Several pathways were shared by all four diseases. Interestingly, HIF-1 (Hypoxia-Inducible Factors 1) signaling pathway and the pathways in cancer are the two KEGG pathways shared by all four diseases (See Venn diagram in Fig. 5a). HIF-1 is a heterodimer, with an alpha subunit regulated by O_2_ and a beta subunit known as aryl hydrocarbon nuclear translocator [54] that plays an important role in cellular adaptation to hypoxia [55]. Since hypoxia is found among people with COPD, pulmonary sarcoidosis and acute lung injury, HIF-1 signaling pathway is important in those diseases [56–58]. It is not surprising that the pathways in cancer also appeared. Both COPD and lung cancer are closely linked diseases because they are both caused by cigarette smoking and are diseases of an aging lung [59]. In addition, pulmonary sarcoidosis and acute lung injury were also reported to be associated with lung cancer [60, 61]. HIF1A and VEGFA (vascular endothelial growth factor A) are the two genes shared by the four diseases in both the HIF-1 signaling pathway and the pathways in cancer. HIF1A is the master transcriptional regulator of cellular and developmental response to hypoxia [62]. VEGFA stimulates endothelial cell mitogenesis and cell migration. Both VEGFA and HIF1A are associated with susceptibility and progression of COPD with HIF1A [63], they also play important roles in pulmonary sarcoidosis [64] and acute lung injury [65].

## Discussion & Conclusions

In this project, we introduced a mathematical model based on the gene to PubMed mapping to characterize a disease, and the performance of this approach was evaluated with a case study of COPD. Applying this model, we analyzed all the diseases in the branch of Lung diseases in MeSH tree, and were able to successfully distinguish the COPD related and non-related diseases.

Our model predicted 4 novel COPD related diseases. Three of these diseases, Acute Lung Injury, Pulmonary Sarcoidosis, and Lung Injury were identified to be closely related to COPD based on gene information (Figs. 2, 3, 5) and literature (Fig. 4). Our analysis has also shown that lung injury, acute lung injury, COPD and pulmonary sarcoidosis are all related to inflammation and injury in lung. However, because acute lung injury is a branch of lung injury, and not all the children of lung injury relate to COPD, the relationship between Lung injury and COPD could be due to the contribution of acute lung injury as a child of Lung injury.

The identified relationship between Bird Fancier’s Lung and COPD only has shallow semantic connections. One possible reason is that the Bird Fancier’s Lung is not extensively studied and there is a lack of sufficient experiment evidence. This is reflected in the fact that no search result returned for the Bird Fancier’s Lung from IPA™ and DAVID—two integrative, widely used annotation databases for genes and diseases. The sensitivity of the Gene Fingerprint approach to detect disease-disease relationships could be a strength for studying diseases with limited experimental data. Further improvement such as replacing Spectral clustering algorithm by deep learning could further improve the performance of our approach in the future using large amount of training/testing data from literature and other sources.

## Abbreviations

COPD: Chronic Obstructive Pulmonary Disease
DAVID: The database for annotation, visualization and integrated discovery
GWAs: Genome wide association study
IPAT^M^: Ingenuity pathway analysis
LSA: Latent semantic analysis
MeSH: Medical subject heading
SemMedDB: Semantic MEDLINE database
SVD: Singular value decomposition
